# Considerations for Phage Therapy Against *Mycobacterium abscessus*

**DOI:** 10.3389/fmicb.2020.609017

**Published:** 2021-01-18

**Authors:** Abrar Senhaji-Kacha, Jaime Esteban, Meritxell Garcia-Quintanilla

**Affiliations:** Department of Clinical Microbiology, IIS-Fundación Jiménez Díaz, Madrid, Spain

**Keywords:** Mycobacterium abscessus, non-tuberculous mycobacteria, phage therapy, alternative therapy, antibiotic resistance, bacteriophage

## Abstract

There is a global increasing number of *Mycobacterium abscessus* infections, especially pulmonary infections. Reduced therapeutic options exist against this opportunistic pathogen due to its high intrinsic and acquired levels of antibiotic resistance. Phage therapy is a promising afresh therapy, which uses viruses to lyse bacteria responsible for the infection. Bacteriophages have been recently administered under compassionate use to a 15-year-old patient infected with *M. abscessus* in combination with antibiotics with excellent results. This mini review highlights different recommendations for future phage administrations such as where to look for new phages, the use of cocktail of mycobacteriophages to broaden phage specificity and to tackle resistance and phage insensitivity due to temperate phages present in bacterial genomes, the combined use of phages and antibiotics to obtain a synergistic effect, the liposomal administration to reach a prolonged effect, intracellular delivery and protection against neutralizing antibodies, and the convenience of using this strategy in patients suffering from cystic fibrosis (CF) since phages are believed to promote immunomodulatory actions and eliminate biofilms.

## Introduction

There is a global concern about the increasing number of multidrug resistant bacteria. The Centers for Disease Control (CDC) and the World Health Organization (WHO) have declared antibiotic resistance a threat to global health. Despite the increasing number of resistant bacteria, there is a decreasing effort in manufacturing new antibiotics from pharmaceutical companies. From 1983 to 1987, 16 new antibiotics were approved by the Food and Drug Administration (FDA) in the United States, however, between 2010 and 2016 only six new antibiotics were authorized (Luepke et al., 2017).

Importantly, *Mycobacterium abscessus* is the most challenging species of mycobacteria due to its intrinsic and acquired bacterial resistance. In the last decade, *M. abscessus* infections have increased including pulmonary infections, systemic and/or disseminated infections, and skin and soft-tissue infections (Johansen et al., 2020). In pulmonary infection, *M. abscessus* causes chronic respiratory diseases in patients suffering from underlying lung diseases, such as cystic fibrosis, bronchiectasis, and chronic obstructive pulmonary disease. This increment is due to a growing number of patients with immunosuppression and increased life expectancy, as well as other factors that predispose to these infections (Chalmers et al., 2018). *Mycobacterium abscessus* present a phenotypic heterogeneity based on the presence (smooth) or absence (rough) of glycopeptidolipids (GPLs) extracellularly, rough derivatives exhibiting higher levels of virulence than smooth variants (Catherinot et al., 2007; García-Coca et al., 2019).

Phage therapy is a promising afresh therapy, which uses viruses infecting bacteria to deal with infections. In 2014, the United States National Institute of Infectious Diseases included phage therapy as one of seven strategies to tackle antibiotic resistance (Reardon, 2014). A cocktail of three bacteriophages has been recently administered under compassionate use to a 15-year-old patient infected with *M. abscessus* in combination with antibiotics. The excellent results obtained show that this approach can help to cure infections against this particular pathogen.

## Current Treatment Against *Mycobacterium Abscessus*

*Mycobacterium abscessus* is a rapidly growing non-tuberculous mycobacteria (NTM) and an opportunistic pathogen. Treatments last from months to years and are associated to potential risky antibiotic toxicity and a high number of failures. This species is composed by three subspecies: *M. abscessus* subsp. *abscessus*, subsp. *bolletii* and subsp. *massiliense* (Tortoli et al., 2016) although its taxonomy remains under debate.

Current recommendations to treat *M. abscessus* depend on subspecies. While subspecies *massiliense* is sensitive to macrolides, subspecies *abscessus* and *bolletii* are usually resistant due to the presence of an inducible methylase encoded by the *erm(41)* gene (Nash et al., 2009).

According to the recommendations found in the official ATS/ERS/ESCMID/IDSA Clinical practice guideline (Daley et al., 2020), a susceptibility-based treatment for macrolides and amikacin is suggested over empiric therapy in patients with *M. abscessus* pulmonary disease. A multidrug regimen consisting of three or more active drugs is recommended. Importantly, a macrolide-containing treatment is recommended not only in patients without inducible or mutational resistance, but also under macrolide resistance if macrolides are used for immunomodulatory reasons. The duration of the therapy will depend on each infection. Additional recommendations can be found in the British Thoracic Society guidelines (Haworth et al., 2017).

Novel therapeutic options, including old drugs, iron chelators, or antimicrobial peptides show different degrees of activity against *M. abscessus* (Bento et al., 2020; Meir and Barkan, 2020; Muñoz-Egea et al., 2020). In this review, we will focus on phage therapy as an alternative therapy against *M. abscessus* multiresistance.

## Phage Therapy as Alternative Therapy

Bacteriophages are viruses that infect bacteria and after infection can be lysogenic or lytic. Therapeutic phage use was discovered a century ago, however, after the development of antibiotics, only Eastern Europe continued using this alternative under compassionate use. The current reintroduction in Western countries is caused by the increasing number of multidrug-resistant bacteria and the scarce number of novel antibiotics. The first patient treated with phage therapy in the US suffered from a systemic infection caused by a multidrug-resistant *Acinetobacter baumannii* and completely recovery was achieved (Schooley et al., 2017).

Recent reviews have shown the efficacy and the state of the art of phage therapy research applied to humans, typically, phages are delivered by intravenous or topical route more than once until bacterial count decrease and clinical improvement is achieved. In 2019, McCallin et al. (2019) highlighted different cases of phage therapy under compassionate use. Patey et al. (2018) reviewed all local compassionate use of phages combined with antibiotics at the Villeneuve Saint Georges Hospital (France), describing complete recovery in 12 out of 15 cases, two cases of improvement without eradication, and one eradication with a secondary pathogen emerging. Recent reviews about clinical trials describe results of phage therapy to treat chronic otitis, infected burn wounds, diarrhea, diabetic foot ulcers, venous leg ulcers, urinary tract infections, or gastrointestinal disorders caused by *Staphylococcus aureus*, *A. baumannii*, *Pseudomonas aeruginosa*, and the *Enterobacteriaceae* family with different results (Furfaro et al., 2018; Altamirano and Barr, 2019), but no clinical trial using bacteriophages against mycobacteria has been performed. Finally, Brix et al. (2020) summarized different animal models used to study phage therapy, including *Caenorhabditis elegans*, *Drosophila melanogaster*, *Galleria mellonella*, zebrafish, quail, chicken, rabbit, hamster, and mouse.

Phage utilization against *Listeria monocytogenes*, *Salmonella enterica* serovar Typhimurium and *Escherichia coli* have been approved for food industry. These products have been named as *Generally Recognized As Safe* (GRAS) by the US Food and Drug Administration (FDA), however, no approval is available for standard therapeutic use to date (Sarhan and Azzazy, 2015).

## Successful Case Report Against *Mycobacterium Abscessus*

More than 10,000 mycobacteriophages have been isolated to date from different sources, and most of the registered phages infect *Mycobacterium smegmatis* (The Actinobacteriophage Database, 2020; Table 1). This database shows just three phages infecting *M. abscessus* GD01 (one sequenced) and one phage infecting *M. abscessus* subsp. *bolletii* F1660. The 17% of total mycobacteriophages have been sequenced and classified into 29 clusters, 71 sub-clusters, and 10 singletons (Sinha et al., 2020). Phages infecting *Mycobacterium* genus are tailed with double-stranded DNA and belong to Siphoviridae and Myoviridae families, which harbor long flexible non-contractile tails and contractile tails, respectively (Hatfull, 2018). Mycobacteriophages have been traditionally utilized as tools for diagnosis or genetic manipulation.

**Table 1:** Registered phages infecting *Mycobacterium* genus.

Species (N strains)	Number of phages	Sequenced phages
*M. abscessus* (2)	3	1
*M. aichiense* (1)	1	1
*M. aurum* (1)	4	0
*M. avium* (7)	9	7
*M. phlei* (2)	8	8
*M. smegmatis* (2)	11,388	1,931
*M.* sp. (1)	1	0
*M. tuberculosis* (1)	1	1

N strains: number of strains (The Actinobacteriophage Database, 2020).

Thirteen phages (DS-6A, TM4, D29, T7, P4, PDRPv, BTCU-I, Bo4, SWUI, GR-2I/T, My-327, Ms6, and Bxz2) were recently reviewed as candidates for phage therapy against *Mycobacterium tuberculosis* and other mycobacteria (Azimi et al., 2019). Another mycobacteriophage (ZoeJ) related to TM4 has been described as a broad host-range infecting *M. tuberculosis*, *M. smegmatis*, and *Mycobacterium avium* (Dedrick et al., 2019a).

The only study published about phage therapy against *M. abscessus* is the case report published in 2019 of a 15-year-old patient suffering from cystic fibrosis (CF) who had a double lung transplant. The patient had been treated for 8 years against chronic *P. aeruginosa* and *M. abscessus* subsp. *massiliense* infections. After bilateral lung transplant, immunosuppressive drugs and intravenous antibiotics were delivered. Reddening of surgical wound was noted 1 week after finishing intravenous administration. *Mycobacterium abscessus* was isolated and intravenous antibacterial antibiotics were restarted (amikacin, imipenem/cilastatin, and tigecycline) together with clofazimine and bedaquiline. Infection evolved and 7 months post-transplant the patient suffered from a disseminated infection due to *M. abscessus* with skin lesions. At this point, phage therapy was considered as compassionate use. Screening of more than 10,000 *M. smegmatis* phages against *M. abscessus* lead to the selection of three bacteriophages (Siphoviridae family). The treatment consisted of a cocktail of the three phages introduced by intravenous route, two of them were genetically engineered to delete repressors and convert temperate phages into lytic phages. Phages were detected in serum 1 day after starting, falling below detection limits 6 days afterward. After starting the phage therapy, no *M. abscessus* was isolated from serum or sputum. Finally, the patient was discharged home with antibiotics and phage administration and no adverse effects were reported. Improvement of lungs, liver, and skin was achieved after 7 months of intravenous infusions of phages every 12 h (10^9^ PFU/dose of each phage) and topical administration of phages daily in combination with multiple antibiotics (amikacin, imipenem/cilastatin, tigecycline, bedaquiline, and clofazimine; Dedrick et al., 2019b). This was the first case of phage therapy using genetically modified phages and the first use in patients against a mycobacterial infection.

A recent report about requests for phage therapy at the Center for Innovative Phage Applications and Therapeutics (IPATH) in the United States shows that from June 1, 2018, to April 30, 2020, there have been 90 outcomes of requests for phage therapy against mycobacteria (47 *M. abscessus*, 23 *M. avium*, seven *Mycobacterium chimera*, seven *M.* species, two *Mycobacterium chelonae*, and one *M. smegmatis*, *Mycobacterium xenopi*, *Mycobacterium bolletii*, and *Mycobacterium genavense*). In a summarizing table, they point that nine lytic phages were found against the isolates of *M. abscessus* and the phage therapy was administered to four patients with a median time from request to administration of 176 days, however, no more information is given about these treatments (Aslam et al., 2020).

There is a lack of preclinical studies about phage therapy against *M. abscessus*; however, current preclinical models of *M. abscessus* infections are diverse. A persister assay for *M. abscessus* has been recently developed (Yam et al., 2020), which could be used to test phage efficacy against persister variants. Notably, Sb-1 phage was able to direct kill *S. aureus* persisters *in vitro* (Tkhilaishvili et al., 2018). Interestingly, the first mouse model of pulmonary *M. abscessus* infection in an immunocompetent mouse strain has been recently described (Maggioncalda et al., 2020). Traditionally, immunocompromised mice have been used for murine infection models due to the rapid clearance of *M. abscessus* by immunocompetent hosts (Obregón-Henao et al., 2015). CF mouse models also exist and would be desirable to test preliminary phage efficacy in CF patients (Guilbault et al., 2007).

## Tips for Phage Therapy Against *Mycobacterium Abscessus*

### Environmental Reservoirs

Phages are ubiquitous in the environment including the human body; nevertheless, every phage will be encountered near its bacterial host. More than 17,000 actinobacteriophages have been described and the genome diversity of the sequenced phages has been studied (Hatfull, 2020). *Mycobacterium abscessus* is able to survive in adverse environments and is found in water sources, soil, and also in sinks, showerheads, or sewage (Lopeman et al., 2019). Novel lytic mycobacteriophages infecting *M. abscessus* should be searched in similar scenarios to directly proceed to the phage enrichment with the clinical bacterium, which is responsible of the infection. Due to phage specificity, tailored treatments are necessary to maximize success of phage therapy, although this personalized manufacture undoubtedly will raise the time from request to administration.

### Cocktail of Phages to Tackle Bacterial Resistance and Insensitivity

Phage resistance in bacteria includes mutations that modify or hide phage receptors, destruction of viral DNA using nucleases or restriction enzymes, and *via* CRISPR-Cas system (Labrie, 2010). Modifications in polysaccharides, teichoic acids, and outer membrane proteins block phage adsorption in Gram-positive bacteria. However, phage-resistant variants are often balanced with bacteria-fitness costs usually associated with decreased virulence or higher susceptibility to antibiotics (Mangalea and Duerkop, 2020), although a faster growth-rate derivative has also been described (Kashiwagi and Yomo, 2011). Temperate phages may represent an obstacle for the entry of invasive phages, the analysis of temperate mycobacteriophages infecting *M. tuberculosis* and *M. smegmatis* exhibited at least five types of defense systems that avoided phage infection (Dedrick et al., 2017), although phages can in turn escape these mechanisms (Hatfull, 2020). Prophages are abundant in bacterial genomes including mycobacteria. A recent study of prophages within clinical NTM concluded that it was more likely to find prophages in rapidly compared to slowly growing bacteria and also suggested that prophages from clinical mycobacteria may contain more virulence genes than environmental mycobacteria (Glickman et al., 2020). Importantly, a genome analysis of 48 *M. abscessus* strains concluded that at least 17 mycobacteriophages have infected *M. abscessus* during its evolution and the authors located one to eight prophage regions in 47 out of the 48 genomes, from intact prophages to small prophage-like elements (Sassi et al., 2014). A single-particle electron microscopy reconstruction of a prophage infecting *M. abscessus* subsp. *bolletii* was reported (64-kb genome; Sassi et al., 2013). To overcome bacterial resistance and the presence of temperate phages in *M. abscessus*, phage therapy should use cocktails of phages which, in addition, broaden the spectrum of infection.

### Antibiotic-Phage Combinations

The mycobacteriophage SWU1 gp39 has been proposed as an enhancer of isoniazid, erythromycin, norfloxacin, ampicillin, ciprofloxacion, ofloxacin, rifampicin, and vancomycin antibiotics against *M. smegmatis via* modification of cell wall permeability (Li et al., 2016, 1). Of special interest is the effect of phages on biofilms after producing enzymes that breakdown the extracellular matrix enabling metabolic revival of bacteria and antibiotic arrival (Chakraborty and Kumar, 2019). Interestingly, subcutaneous administration of lysin B (a D29 enzyme) was able to prevent bacteria proliferation in a mouse model of *Mycobacterium ulcerans* footpad infection (Fraga et al., 2019). These combinations could diminish doses and timing of antibiotics against *M. abscessus* reducing adverse effects and toxicity of long treatments.

### Liposomal Administration

Mycobacteriophages are able to target extracellular bacteria; however, intracellular variants inside macrophages are difficult to access. Encapsulated phages into liposomes are promising for different reasons such as prolonged effect, intracellular delivery, protection against neutralizing antibodies, and preservation from stomach acidic pH in case of oral delivery (Colom et al., 2015; Malik et al., 2017). TM4 mycobacteriophages encapsulated into giant liposomes were shown to enter eukaryotic cells more efficiently than free phages (Nieth et al., 2015). Interestingly, inhaled liposomal amikacin have been evaluated in a randomized phase II clinical trial for NTM-lung-infected patients and results show improvements in sputum conversion (Olivier et al., 2017). One important issue for phage-therapy efficacy is the accessibility of the pathogen to the phages. For this reason, diverse deliveries should be used depending of the site of infection. In case of pulmonary infection, an inhalation delivery could be administered. A comparative study in the delivery rate of D29 phage against *M. tuberculosis* revealed that vibrating mesh nebulizer was more efficient than soft mist inhaler and jet nebulizer, indicating that vibrating mesh nebulizer would be recommended to deliver large amounts of phage (Carrigy et al., 2017). Intravenous phage administration would be desirable to deliver phages throughout the body in systemic and/or disseminated infections caused by *M. abscessus*, and in case of skin and soft tissue infections local application of phages, including intramuscular, subcutaneous, or topical administration should be studied.

### Preferential Use in Patients Suffering From Cystic Fibrosis

Persistent infections in CF patients after lung transplant can result in significant morbidity and mortality (Furukawa and Flume, 2018). *Mycobacterium abscessus* has been detected in 7% of CF patients in United Kingdom (UK Cystic Fibrosis Registry, 2020). CF patients present increased levels of nuclear factor kB (NF*κ*B) with chronic inflammation (Bodas, 2010), and also aberrant trafficking and degradation of Toll-like receptor 4 (TLR4), thereby enhancing inflammatory response (Bruscia et al., 2011). Moreover, rough variants of *M. abscessus* enhance IFN-I and IL-1*β* production *via* mitochondrial ROS (Kim et al., 2020). Phages are believed to promote immunomodulating and anti-inflammatory actions, are able to diminish ROS *in vitro* (Górski et al., 2020) and reduce NFκB activation (Górski et al., 2006). Mice fed with T7 phage showed only a slight increase in IL-17A compared to control mice (Park et al., 2014). Surprisingly, another study reported IFN gamma induction by STA-8505 phage, which infects *S. aureus* (Pincus et al., 2015), indicating that more *in vivo* studies are needed to clarify this point. Remarkably, phages are more abundant in mucosal surfaces *via* binding between mucin glycoproteins and Ig-like protein domains of phages protecting the underlying epithelium from bacterial infection (Barr et al., 2013); this characteristic may increase the multiplicity of infection against the target bacteria in lungs of CF patients. Finally, the biofilm destruction by phages could represent an additional advantage to deal with pulmonary infections efficiently in CF patients.

## Concluding Remarks

*Mycobacterium abscessus* infections require novel approaches. Phage therapy combined with antibiotics represents a promising alternative against *M. abscessus* due to the expected synergistic effect of phages with antibiotics against planktonic bacteria and biofilms. The studies reviewed here lead to considering some productive future research to answer remaining questions about phage therapy against *M. abscessus*: Can cocktails of phages overcome narrow host range and phage resistance against *M. abscessus*? Or is it only possible as tailor-made, personalized therapy? Which antibiotic-phage combination will be the most effective against *M. abscessus*? Can antibiotics combined with mycobacteriophages achieve higher effect with lower doses and timing, reducing adverse effects associated to prolonged use? Which route of administration will be adequate in each type of *M. abscessus* infection (pulmonary, systemic and/or disseminated infections, skin and soft-tissue infections)? Can intracellular *M. abscessus* be eliminated using phages embedded into liposomes? To what extent will phages destroy biofilms in patients with inflammatory lung diseases, such as cystic fibrosis, bronchiectasis, and chronic obstructive pulmonary disease? Specific mouse models of these infections exist. Are limitations of these murine models a big deal? Which type of patients would benefit from a prophylactic and therapeutic phage administration? There are still limitations that should be addressed prior to apply this approach into the clinical practice; however, phage therapy could be currently considered a realistic option under compassionate use.

## Author Contributions

MG-Q and JE conceived the manuscript. AS-K, MG-Q, and JE critically reviewed the literature. AS-K and MG-Q wrote the manuscript. JE revised the manuscript. All authors contributed to the article and approved the submitted version.

### Conflict of Interest

The authors declare that the research was conducted in the absence of any commercial or financial relationships that could be construed as a potential conflict of interest.
