# Corrigendum: Considerations for Phage Therapy Against *Mycobacterium abscessus*

**DOI:** 10.3389/fmicb.2021.722831

**Published:** 2021-07-14

**Authors:** Abrar Senhaji-Kacha, Jaime Esteban, Meritxell Garcia-Quintanilla

**Affiliations:** Department of Clinical Microbiology, IIS-Fundación Jiménez Díaz, Madrid, Spain

**Keywords:** *Mycobacterium abscessus*, non-tuberculous mycobacteria, phage therapy, alternative therapy, antibiotic resistance, bacteriophage

In the original article, we neglected to include the funders Instituto de Salud Carlos III and European Union, European Social Fund “Investing in your future” (CP19/00104) to MG-Q. The corrected Funding statement appears below.

MG-Q is supported by the Subprograma Miguel Servet from the Ministerio de Ciencia e Innovación of Spain, the Instituto de Salud Carlos III and the European Union, European Social Fund “Investing in your future” (CP19/00104).

The authors apologize for this error and state that this does not change the scientific conclusions of the article in any way. The original article has been updated.

